# Echo- and Image-Domain Fusion for Micro-Leak Detection and Localization in Subsea Gas Pipelines

**DOI:** 10.3390/s26165142

**Published:** 2026-08-14

**Authors:** Haichao Liu, Jian Li, Xiaobin Jiang, Yi Luo

**Affiliations:** 1 School of Precision Instrument and Optoelectronics Engineering, Tianjin University, Tianjin 300072, China; liuhch@cnooc.com.cn (H.L.); tjupipe@tju.edu.cn (J.L.); 2 CNOOC (Tianjin) Pipeline Engineering Technology Co., Ltd., Tianjin 300450, China; jiangxb3@cnooc.com.cn; 3 Tianjin Key Laboratory of Subsea Pipeline, Tianjin 300450, China; 4 CNOOC Energy Technology & Service Limited Key Laboratory for Intelligent Manufacturing and O&M of Offshore Equipment, Tianjin 300450, China

**Keywords:** subsea pipeline, micro-leakage detection and localization, acoustic imaging, echo-image domain fusion

## Abstract

**Highlights:**

**What are the main findings?**

- Controlled offshore experiments demonstrate that the system maintains reliable detection even under extreme micro-leak conditions (0.5 mm aperture, 0.5 MPa), indicating high sensitivity to incipient leaks.

**What are the implications of the main findings?**

- The independently developed dedicated multibeam forward-looking sonar instrument breaks through the sensitivity bottleneck of sonar systems in micro-leak detection scenarios.

**Abstract:**

The detection of microleaks in subsea gas pipelines remains challenging in shallow-water environments because weak bubble-plume echoes are often obscured by seabed reverberation, ambient noise, and platform-induced interference. This study develops a custom multibeam forward-looking sonar system and a dual-domain framework for acoustic detection and localization of underwater gas microleakage. Local statistical enhancement suppresses stable background interference and strengthens anomalous bubble echoes. Blind deconvolution, adaptive grid-based thresholding, and spatial clustering then improve target sharpness, extract candidate bubble-plume regions, and reduce localized false detections. This unsupervised framework requires no pre-collected training data. It was evaluated in 30 independent sea-trial groups conducted in Bohai Bay using air to simulate leakage. Six orifice diameters from 0.5 to 3.0 mm were tested under different compressor-indicated pressures. The plume-observation distances (defined as the sonar-to-leak-device distance at the first confirmed plume response in the sonar image) ranged from 32 to 66 m across the tested conditions. Under the minimum tested condition of a 0.5 mm orifice and a compressor-indicated pressure of 0.5 MPa, the plume was observed at a distance of 32 m. The results demonstrate the feasibility of the proposed system for ROV-assisted detection and localization of subsea gas microleakage in low-visibility shallow-water environments.

## 1. Introduction

Subsea pipelines are essential infrastructure for offshore oil and gas transportation, and their integrity directly affects operational safety, economic performance, and marine environmental protection. Pipeline leakage can lead to product loss, production interruptions, equipment damage, and long-term ecological consequences. Among the various leakage scenarios, microleaks are particularly difficult to detect because gas escaping through a small aperture usually generates weak, localized, and sometimes intermittent signatures. These signatures may remain undetected for an extended period while the defect continues to deteriorate. Reliable detection of incipient leakage is therefore essential for condition-based maintenance, risk mitigation, and the sustainable operation of subsea production systems [1,2].

Existing subsea pipeline leak detection techniques can generally be classified as pressure- and flow-based monitoring, optical inspection, fiber-optic sensing, passive acoustic sensing, and active acoustic imaging [3,4,5,6,7,8]. Pressure- and flow-based methods are widely used for continuous monitoring because they can be integrated into existing supervisory and control systems. However, small and localized leaks may produce only minor variations in operational parameters, which can be obscured by normal fluctuations in pressure and flow [3,4]. Optical inspection provides direct visual evidence of gas release but is strongly affected by water turbidity, insufficient illumination, suspended particles, and limited underwater visibility [5]. Distributed fiber-optic sensing can provide continuous and spatially resolved measurements along a pipeline, but it generally requires dedicated installation and close physical integration with the monitored infrastructure. Consequently, its deployment may be costly and technically challenging for existing subsea pipeline networks [6,7,8].

Underwater acoustic sensing provides a promising alternative because sound waves can propagate over much greater distances than light in turbid marine environments. Acoustic methods for subsea leak detection can be broadly categorized into passive and active approaches, each with distinct capabilities and limitations. Passive acoustic methods exploit the self-generated acoustic emissions of escaping gas. Yang et al. [4] demonstrated source-bearing estimation using a single-vector hydrophone, and others have investigated leakage-sound characterization and weak-signal enhancement [9,10,11]. The principal limitation of passive sensing, however, is its reliance on the leakage source producing sufficiently strong and distinguishable acoustic emissions—a condition that is often not met for micro-leaks, where bubble formation and release generate only weak, intermittent signals that are easily masked by ambient noise. Active imaging sonar addresses this limitation by transmitting acoustic pulses and analyzing the backscattered echoes, thereby obtaining both echo intensity and spatial information [12,13,14]. Within this category, multibeam forward-looking sonar is particularly attractive for mobile subsea inspection because of its wide field of view, high update rate, and ability to operate under low-visibility conditions. When integrated with ROVs or AUVs, it can support flexible inspection of extensive or inaccessible pipeline sections [15,16,17]. Previous studies have demonstrated the feasibility of using multibeam sonar images to identify bubble plumes [12], recognize underwater objects [9,10], and classify gas-release conditions [14].

However, most existing active-sonar approaches operate exclusively in the image domain—that is, they perform detection and classification after the acoustic data have been beamformed and converted into a visual image. This paradigm works well for targets with strong echo returns and clear spatial structure, but faces significant challenges for micro-leak detection. As noted by several researchers [13,18], weak bubble plumes exhibit low echo energy, irregular morphology, and poor spatial continuity, making them difficult to distinguish from seabed reverberation and background clutter in sonar images. Critically, the beamforming and image-formation process discards or attenuates statistical information present in the raw echo domain—such as local amplitude fluctuations and temporal variability—that could provide valuable cues for distinguishing bubble signatures from stationary reverberation. Only a few studies have begun to exploit echo-domain statistical features for gas detection [18], and to our knowledge, no prior work has systematically combined both echo-domain statistical enhancement and image-domain structural restoration in a unified framework for subsea micro-leak detection. Comparison of representative methods for subsea gas leak detection and related acoustic processing are summerised in Table 1.

Despite these advances, reliable detection of gas microleakage in shallow water remains challenging. Ambient noise, multipath propagation, seabed reverberation, suspended-particle scattering, and platform-induced interference can mask the weak acoustic signatures generated by small bubble plumes [9,12,18]. Moreover, microleak plumes typically exhibit low echo energy, irregular morphology, and poor spatial continuity, making them difficult to distinguish from reverberation and isolated background clutter. Most existing methods focus primarily on interpreting sonar images after beamforming. Although image-domain processing can enhance target contours and extract spatial structures, some statistical information contained in the original acoustic echoes may be weakened or lost during image formation. In particular, local differences in amplitude distribution and temporal stability among bubble echoes, background reverberation, and ambient noise may provide valuable cues for enhancing weak leakage signatures before acoustic imaging. However, relatively few studies have systematically combined echo-domain statistical information with image-domain structural features for subsea gas microleak detection and localization [11,12,13,14,18].

**Figure 1:**
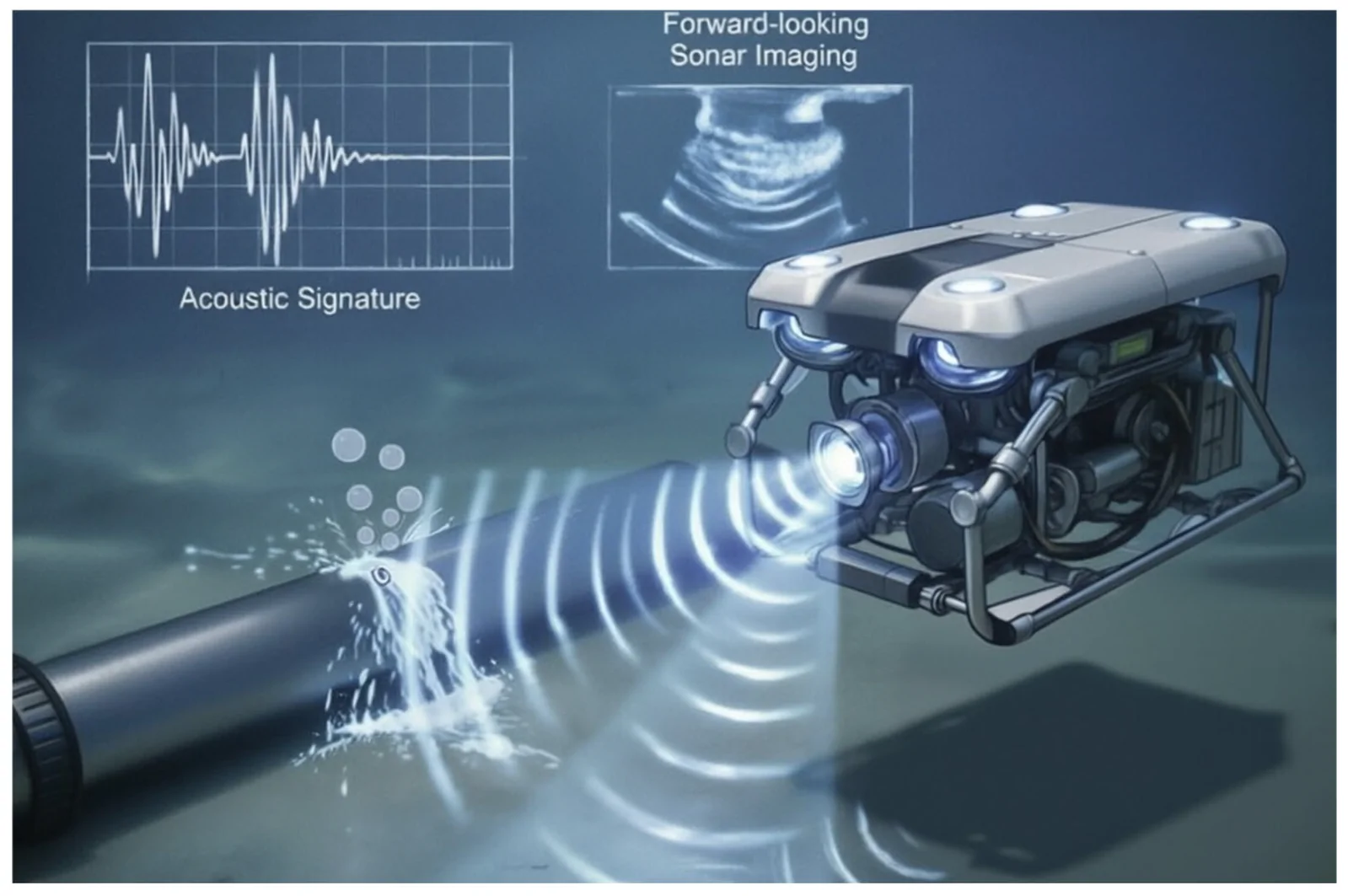
Overview of the proposed multibeam forward-looking sonar system for subsea gas microleak detection.

To address these limitations, this study develops a dedicated multibeam forward-looking sonar system and an unsupervised echo- and image-domain processing framework for mobile detection and localization of subsea gas microleakage in shallow water. An overview of the proposed sonar-based microleak inspection system, including its operating scenario, underwater electronic architecture, and representative sea-trial performance, is presented in Figure 1.

The sonar platform comprises a 96-element horizontal receiving array, an arc-shaped transmitting array, an underwater data-acquisition unit, and a topside processing platform, providing the necessary spatial coverage and angular resolution for bubble-plume imaging. On the basis of this platform, a dual-domain processing method is developed to exploit the complementary characteristics of raw acoustic echoes and formed sonar images. Local statistical enhancement is first applied in the echo domain to suppress stable background interference and strengthen anomalous bubble-plume returns. The enhanced echoes are then transformed into acoustic images, in which blind deconvolution, adaptive grid-based thresholding, and spatial clustering are sequentially performed to improve target sharpness, extract candidate plume regions, and remove isolated false detections. Because the proposed framework does not require pre-collected training samples, it avoids the strong dependence of supervised learning methods on large, labeled, and environment-specific datasets.

The proposed system was evaluated through 30 independent sea-trial groups conducted in Bohai Bay, where compressed air was released underwater to simulate subsea gas leakage. Six release-orifice diameters ranging from 0.5 to 3.0 mm were tested under different compressor-indicated pressures. The measured maximum detectable distances ranged from 32 to 66 m across the tested leakage conditions. Under the minimum tested condition, corresponding to an orifice diameter of 0.5 mm and a compressor-indicated pressure of 0.5 MPa, the bubble plume was detected at a maximum distance of 32 m. These results demonstrate the feasibility of combining a dedicated multibeam forward-looking sonar platform with dual-domain signal processing for detecting weak gas-release signatures in reverberant shallow-water environments.

## 2. Acoustic Imaging Instrument and Methodology

### 2.1. Overall Architecture of the Underwater Acoustic Imaging Instrument

**Figure 2:**
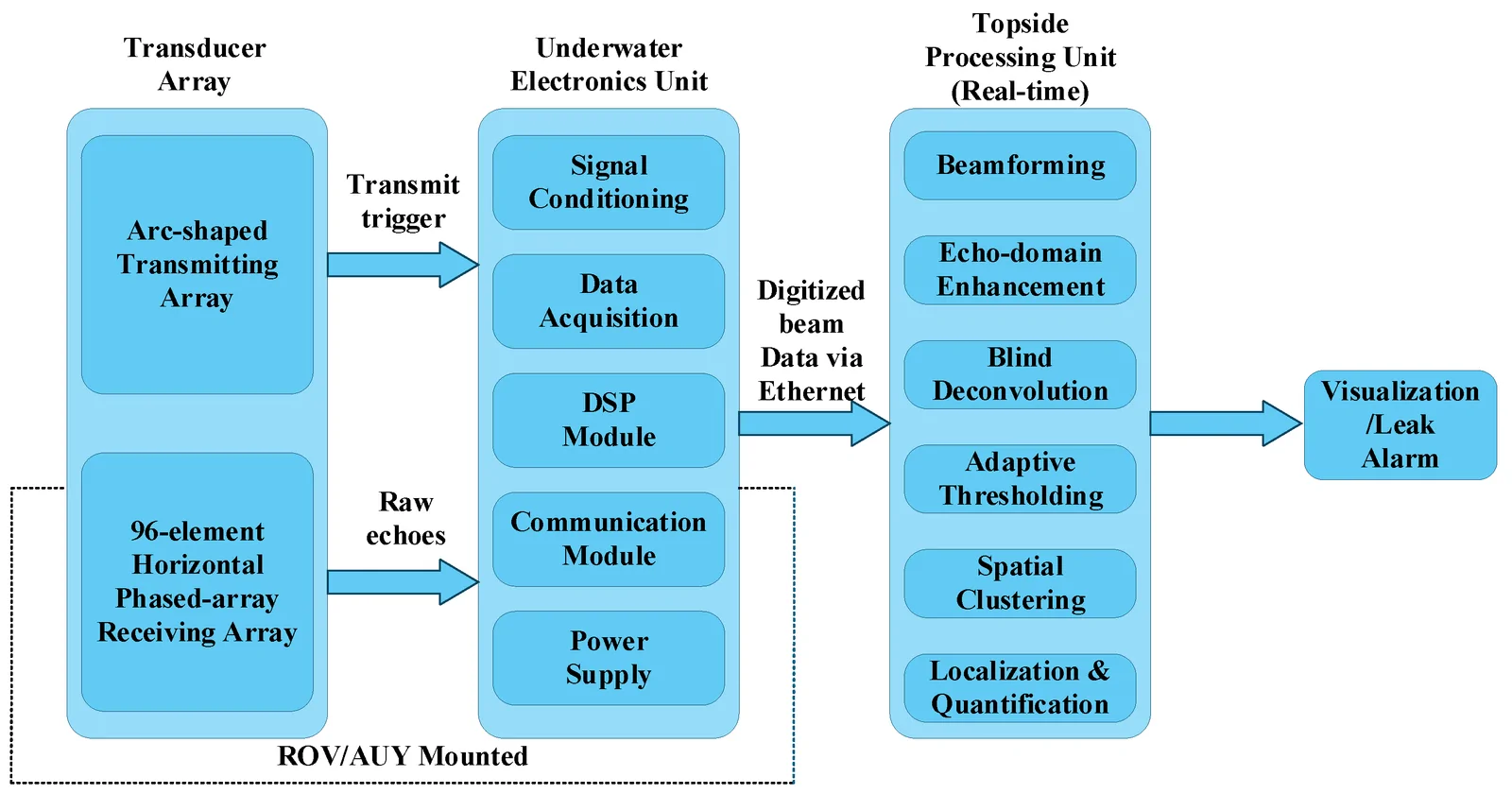
System block diagram of the proposed instrument.

To acquire weak acoustic returns generated by subsea gas micro-leaks, a multibeam forward-looking sonar instrument was developed for mobile pipeline inspection. The system was designed to detect spatially discontinuous bubble-plume echoes in shallow-water environments, where seabed and surface reverberation, ambient noise, multipath propagation, and structural clutter can substantially reduce target-to-background contrast. As shown in Figure 2, the instrument consists of three main components: an acoustic transducer array, an underwater electronics unit, and a topside processing and display module.

The hardware configuration was selected according to the complementary requirements of spatial coverage and angular discrimination. Specifically, the transmitting array provides broad forward-looking insonification, whereas the multielement receiving array forms narrow horizontal beams to distinguish localized bubble-plume returns from pipeline and seabed responses. The acquired multichannel signals are first converted into a range–angle acoustic map through beamforming. Because beamforming alone cannot completely suppress spatially nonuniform reverberation or compensate for the finite point-spread response of the system, the resulting map is subsequently processed using the echo- and image-domain procedures described in Section 2.2, Section 2.3 and Section 2.4.

The acoustic front end comprises a 96-element horizontal phased-array receiving transducer and an arc-shaped transmitting array. The receiving array forms multiple narrow beams in the horizontal plane, providing the angular discrimination required to separate potential bubble-plume returns from pipelines and surrounding clutter. The arc-shaped transmitting array insonifies a broad forward-looking sector and therefore provides the acoustic coverage required for mobile patrol inspection.

**Figure 3:**
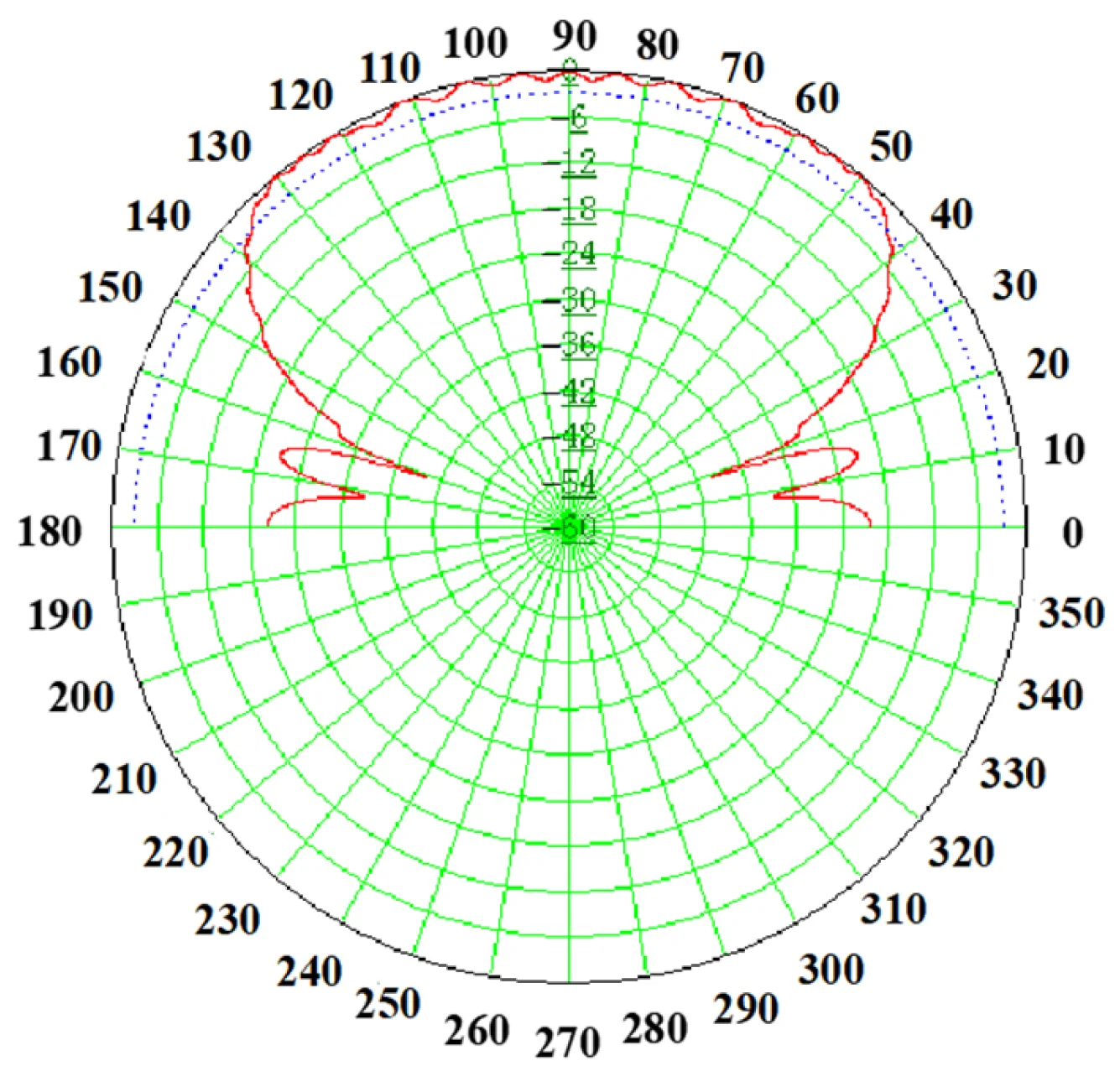
Horizontal directivity diagram of arc-shaped launch array.

**Figure 4:**
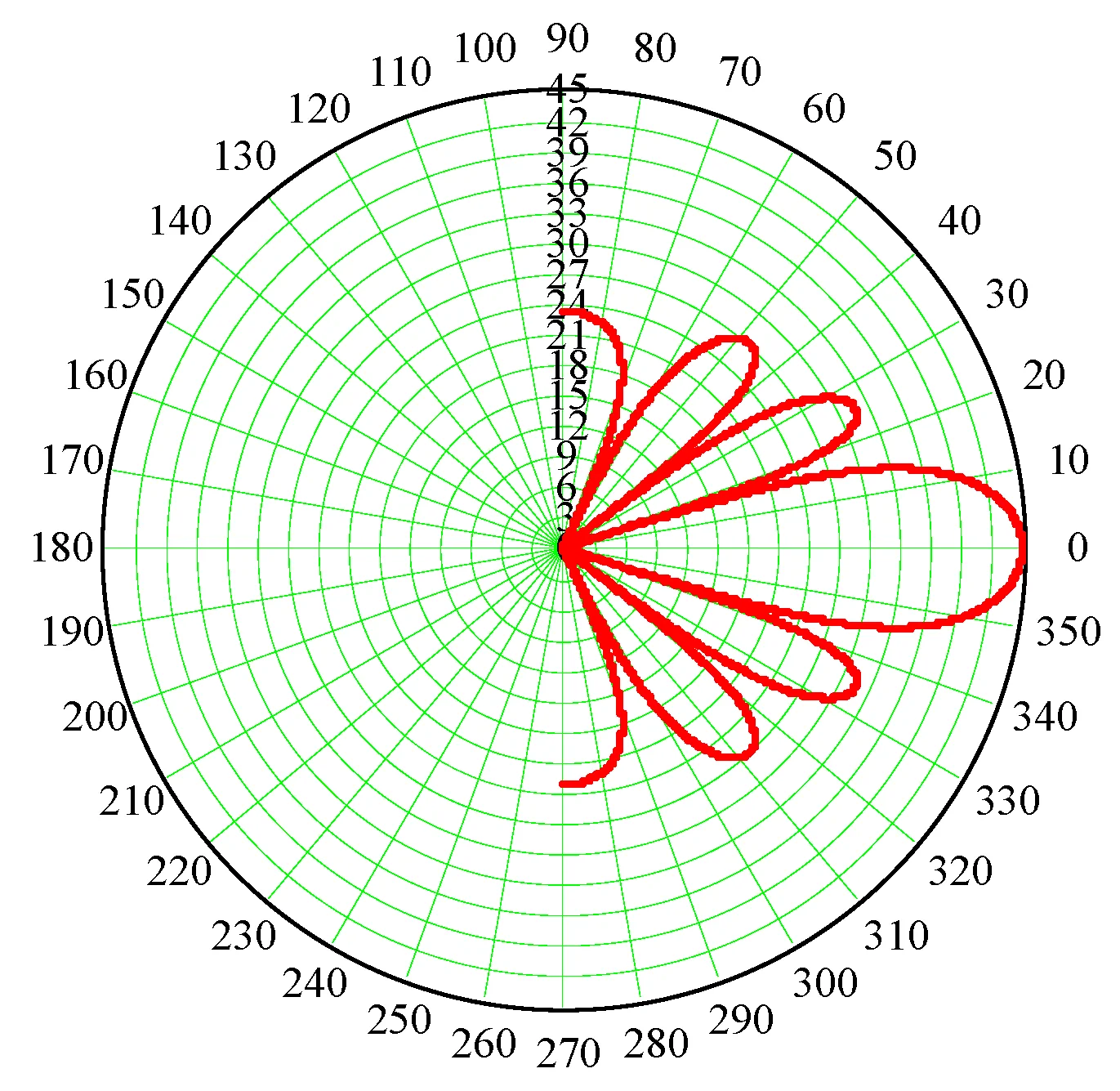
Vertical directivity diagram of arc-shaped launch array.

The horizontal directivity of the arc-shaped transmitting array, which has a radius of 70 mm and a central angle of $90^{\circ }$, is shown in Figure 3. Its measured −3 dB beamwidth is $92.9^{\circ }$, providing broad horizontal coverage of the inspection region. The vertical directivity, which is primarily determined by the effective element length of 5.5 mm, is presented in Figure 4. The transmitting array provides a vertical −3-dB beamwidth of $15.2^{\circ }$ and a first-sidelobe level of −13 dB, allowing most of the transmitted acoustic energy to be concentrated within the intended vertical inspection sector.

**Figure 5:**
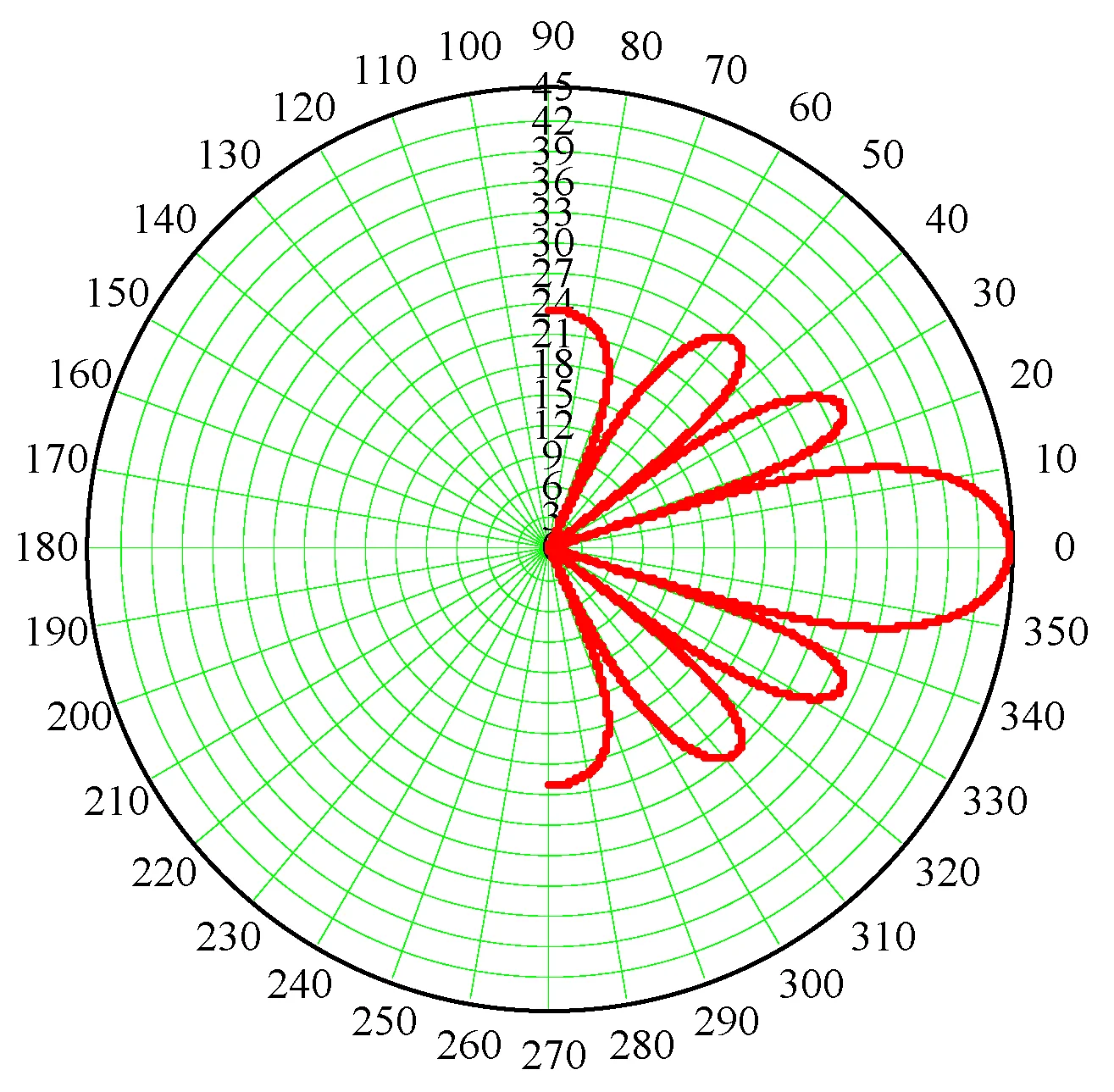
Vertical directivity diagram of receiving array.

**Figure 6:**
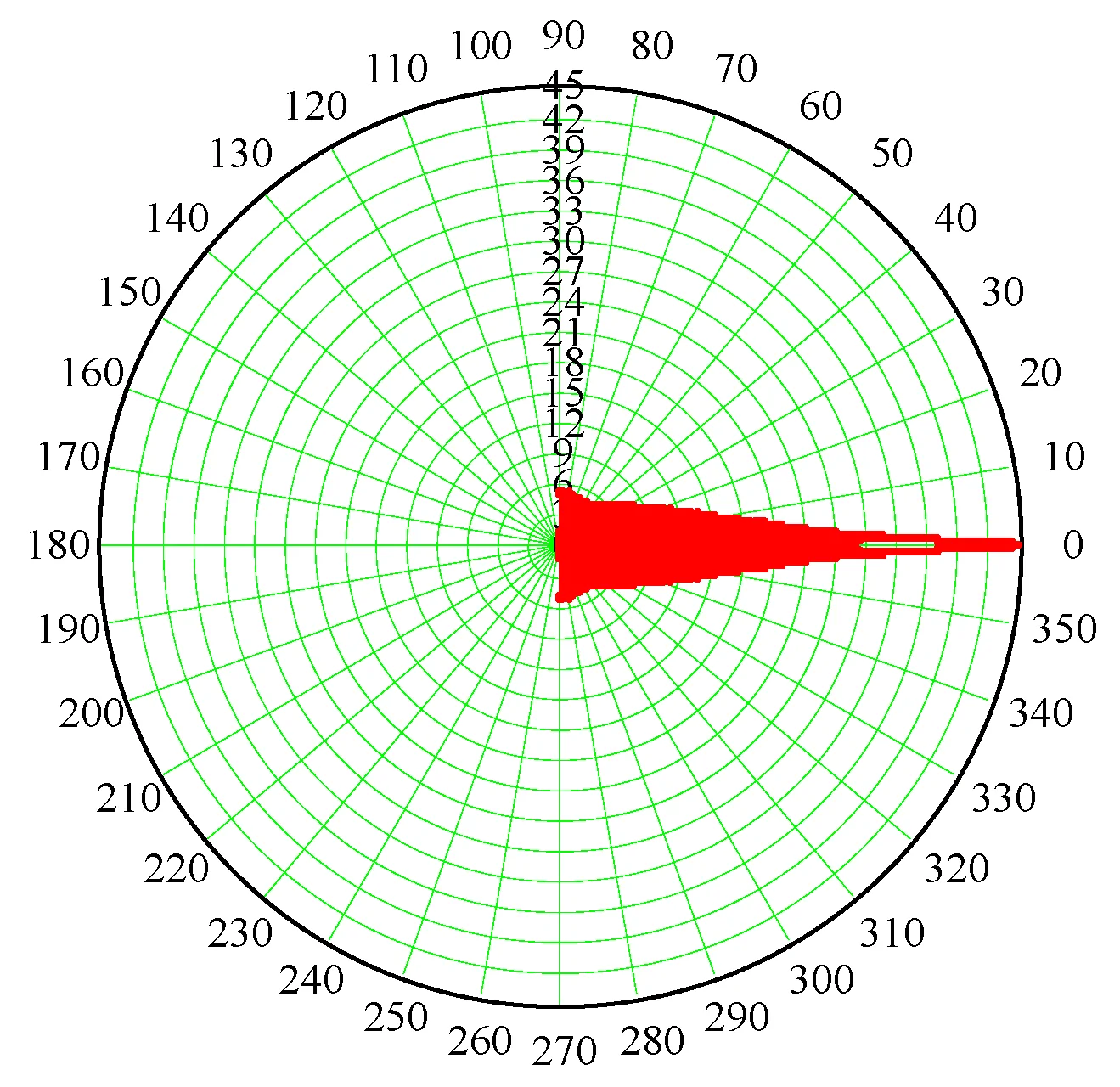
Horizontal directivity map of receiving array.

For reception, the vertical directivity of the 96-element linear array, whose elements also have an effective length of 5.5 mm, is shown in Figure 5. The receiving array provides a vertical −3 dB beamwidth of $15.2^{\circ }$ and a first-sidelobe level of −13 dB. Its horizontal directivity, with an interelement spacing of 0.8 mm, is shown in Figure 6. In the broadside direction, corresponding to a steering angle of $0^{\circ }$, the receiving array achieves a horizontal −3 dB beamwidth of approximately $1^{\circ }$. The effective cross-range resolution nevertheless varies with target range and is further affected by the signal bandwidth, beamforming method, signal-to-noise ratio, and propagation environment.

The underwater electronics unit integrates acoustic signal transmission, multichannel echo acquisition, synchronization control, data buffering, and data-communication modules. During operation, the transmitting module generates acoustic pulses according to a predefined sequence. Echoes scattered by the pipeline, seabed, gas bubbles, and surrounding environment are received by the phased array and subsequently amplified, filtered, digitized, buffered, and transmitted to the topside processing unit.

**Figure 7:**
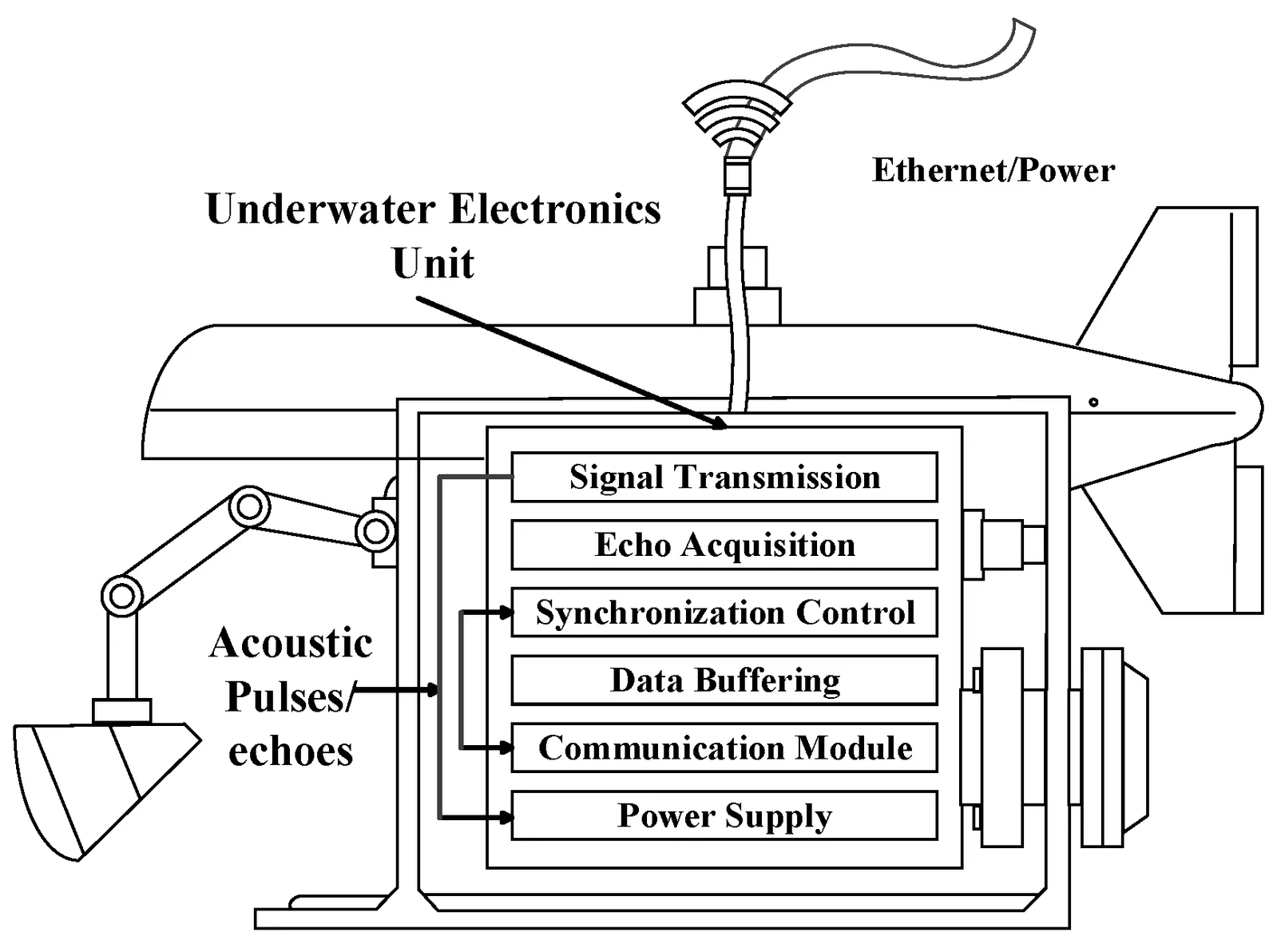
Schematic of the underwater electronics unit mounted on the ROV.

The topside module performs multichannel beamforming, acoustic-map generation, echo- and image-domain processing, candidate detection, sonar-relative localization, feature extraction, and visualization. The instrument can be integrated with a remotely operated vehicle (ROV) or an autonomous underwater vehicle (AUV) for mobile subsea pipeline inspection. Figure 7 illustrates the underwater electronics unit mounted on the experimental ROV.

### 2.2. Multibeam Forward-Looking Sonar Imaging Principle

Let $s_{m}\left(t\right)$ denote the echo signal received by the mth array element, where m=1,2,…,M and M=96. For a steering angle θ, the delay-and-sum beamforming output is expressed as:

(1) $$y_{\theta }\left(t\right)=\sum_{m=1}^{M}w_{m}\;s_{m}\left(t-\tau_{m}\left(\theta \right)\right)$$

where $w_{m}$ is the weighting coefficient applied to the mth element and $\tau_{m}\left(\theta \right)$ is the steering delay determined by the propagation-path difference for direction θ. Taylor or Chebyshev weighting can be applied to reduce the array sidelobes. By electronically scanning a set of steering angles within the forward-looking sector, typically from $-45^{\circ }$ to $45^{\circ }$, the 96-channel time-domain signals are converted into a two-dimensional range–angle representation.

Assuming a homogeneous propagation medium, the range corresponding to fast-time sample t is calculated as

(2) $$r=\frac{ct}{2}$$

where c is the sound speed in seawater and the factor of 2 accounts for the two-way propagation path. A value of $1500\;m\;s^{-1}$ was used in both the beamforming/range conversion and measurement obtained using instrument and model. The uncertainty caused by variations in sound speed is considered in the localization analysis.

After beamforming, the analytic signal is obtained using the Hilbert transform. The acoustic intensity of each range–angle cell is calculated as:

(3) $$I\left(r,\theta \right)={\left|y_{\theta }\left(t\right)+jH\left\{y_{\theta }\left(t\right)\right\}\right|}^{2}$$

where H{⋅} denotes the Hilbert transform and j is the imaginary unit. The resulting intensity matrix forms the initial range–angle acoustic map, which can subsequently be displayed in polar coordinates or transformed into Cartesian coordinates for visualization and image-domain processing.

In a shallow-water environment, the beamformed echo at a given steering angle can be decomposed as:

(4) $$y_{\theta }\left(t\right)=y_{T,\theta }\left(t\right)+y_{R,\theta }\left(t\right)+y_{N,\theta }\left(t\right)+y_{I,\theta }\left(t\right)$$

where $y_{T,\theta }\left(t\right)$ represents target-related echoes from the pipeline or gas bubble plume, $y_{R,\theta }\left(t\right)$ denotes reverberation from the seabed, water surface, and surrounding boundaries, $y_{N,\theta }\left(t\right)$ represents ambient and receiver noise, and $y_{I,\theta }\left(t\right)$ denotes impulsive or platform-related interference.

Although weak bubble-plume echoes may be partially masked by reverberation and may not be visually prominent in the initial acoustic map, they can exhibit localized intensity anomalies and statistical characteristics that differ from those of the surrounding background. These differences provide the basis for the local statistical processing applied before image-domain restoration.

For a local window $\Omega_{r,\theta }$ centered at (r,θ), the local mean and standard deviation are calculated as:

(5a) $$\mu_{\Omega }\left(r,\theta \right)=\frac{1}{|\Omega |}\sum_{(r^{\prime },\theta^{\prime })\in \Omega }I(r^{\prime },\theta^{\prime })$$

(5b) $$\sigma_{\Omega }^{2}\left(r,\theta \right)=\frac{1}{|\Omega |}\sum_{(r^{\prime },\theta^{\prime })\in \Omega }[I(r^{\prime },\theta^{\prime })-\mu_{\Omega }(r^{\prime },\theta^{\prime }){]}^{2}$$

Leakage-related regions generally exhibit locally enhanced intensity and greater spatial variation compared with slowly varying background reverberation. Accordingly, the normalized enhancement map is defined as:

(6) $$E\left(r,\theta \right)=\frac{I(r,\theta )-\mu_{\Omega }(r,\theta )}{\sigma_{\Omega }(r,\theta )+\varepsilon }$$

where ε is a small positive constant introduced to prevent numerical instability when the local standard deviation approaches zero.

Equation (6) is applied to the beamformed two-dimensional range–angle acoustic map I(r,θ), rather than directly to the individual 96-channel time-domain signals $s_{m}\left(t\right)$. Accordingly, “echo-domain enhancement” in this study refers to statistical processing of the beamformed acoustic amplitude or intensity data before image restoration and segmentation. This operation reduces slowly varying local background components while retaining cells whose intensities differ substantially from those of their surrounding neighborhoods.

### 2.3. Echo-Domain Enhancement and Image Restoration

Following the local statistical enhancement described in Section 2.2, the enhanced range–angle map E(r,θ) is converted into an image-domain representation. Because E(r,θ) may contain negative values, whereas Richardson–Lucy deconvolution requires non-negative inputs, the input image is defined as:

g(x,y)=max{T,[E(r,θ)]0}

where T{⋅} denotes the coordinate transformation from the polar range–angle grid to the Cartesian image grid. If restoration is performed directly on the polar grid, T{⋅} represents only intensity normalization and resampling.

Despite echo-domain enhancement, the sonar image may remain spatially blurred because of the finite array beamwidth and the system point spread function (PSF). In shallow-water environments, multipath propagation and reverberation from the seabed and water surface can further reduce target contrast. To mitigate this degradation and improve the separability of weak bubble-plume returns from background clutter, blind deconvolution is applied [15].

The observed sonar image is modeled as

(7) g(x,y)=f(x,y)∗h(x,y)+n(x,y)

where g(x,y) is the observed enhanced image, f(x,y) is the latent image, h(x,y) is the PSF, n(x,y) represents residual noise and modeling errors, and ∗ denotes two-dimensional convolution. Within the local processing region of a candidate plume, the PSF is assumed to be approximately spatially invariant.

The initial PSF $h_{0}\left(x,y\right)$ is constructed from the theoretical horizontal beam pattern of the receiving array. It is constrained by

$$h(x,y)\geq 0,\sum_{x,y}h(x,y)=1.$$

Unlike conventional fixed-PSF Richardson–Lucy restoration, the present blind implementation alternately updates both the latent image and the PSF. The corresponding estimation problem is expressed as:

(8) $$\left(\hat{f},\hat{h}\right)=\arg\underset{f,h}{min}\;D_{KL}\left(g∥f*h\right),subject\;to\;f\geq 0,h\geq 0,\sum_{\left(x,y\right)}h(x,y)=1$$

where $D_{KL}\left(\cdot ∥\cdot \right)$ is the generalized Kullback–Leibler divergence:

(9) $$D_{KL}\left(g∥f*h\right)=\sum_{\left(x,y\right)}\left[g(x,y)ln\frac{g(x,y)}{\left(f*h\right)(x,y)+\varepsilon }-g(x,y)+\left(f*h\right)(x,y)\right]$$

At iteration i, the latent image and PSF are alternately updated as:

(10a) $$f_{i+1}\left(x,y\right)=f_{i}\left(x,y\right)\left\{h_{i}^{†}*\left[\frac{g}{\left(f_{i}*h_{i}\right)+\varepsilon }\right]\right\}\left(x,y\right)$$

(10b) $$h_{i+1}\left(u,v\right)=h_{i}\left(u,v\right)\left\{f_{i+1}^{†}*\left[\frac{g}{\left(f_{i+1}*h_{i}\right)+\varepsilon }\right]\right\}\left(u,v\right)$$

(10c) $$h_{i+1}\left(u,v\right)=\frac{h_{i+1}^{\%}\left(u,v\right)}{\sum_{u,v}h_{i+1}^{\%}\left(u,v\right)}$$

where $h_{i}^{†}(x,y)=h_{i}(-x,-y)$ and $f_{i+1}^{†}(x,y)=f_{i+1}(-x,-y)$ denote the spatially reversed PSF and image, respectively. All multiplications and divisions outside the convolution operation are performed elementwise, and ε prevents division by zero.

At each iteration, the ratio between the observed image and the image predicted by the current estimates,

$$\frac{g}{f_{i}*h_{i}+\varepsilon },$$

provides a multiplicative correction for the latent image. This correction is back-projected using the reversed PSF $h_{i}^{†}$. The PSF is subsequently updated using the newly estimated image $f_{i+1}$, constrained to its prescribed support, and normalized to unit sum. Therefore, Equations (8) and (10) consistently describe a **blind Richardson–Lucy procedure**, rather than a fixed-PSF Richardson–Lucy algorithm.

The procedure is initialized using $f_{0}=g$, while $h_{0}$ is obtained from the theoretical horizontal receiving-beam pattern. The iteration number was empirically fixed at 30 based on a pre-trial dataset that was independent of the final evaluation data. This value produced an acceptable compromise between spatial sharpening and noise amplification for the present instrument and experimental setting; it is not claimed to be universally optimal for other sonar systems or environments.

No explicit regularization term is introduced in Equation (8). Instead, early termination, non-negativity, PSF support restriction, and PSF normalization act jointly as practical constraints on the blind restoration. The final restored image is defined as

$$\hat{f}(x,y)=f_{30}(x,y).$$

The restored image is subsequently used for adaptive segmentation and localization. Its contribution to the complete processing pipeline should be evaluated through the baseline and ablation comparisons reported in Section 3, including at least the following configurations: image-domain processing alone, echo-domain enhancement alone, restoration without adaptive segmentation, and the complete fusion pipeline.

In summary, the restoration step described in this section follows a blind Richardson–Lucy formulation, in which both the latent sharp image and the unknown system PSF are updated alternately, rather than assuming a fixed, pre-measured PSF.

### 2.4. Adaptive Segmentation, Localization, and Feature Extraction

Shallow-water sonar images generally exhibit spatially nonuniform backgrounds because of range-dependent transmission loss, seabed and surface reverberation, and variations in the receiving beam pattern. Consequently, a single global threshold may either suppress weak bubble-plume echoes or generate excessive false detections in regions with strong clutter. To account for these spatial variations, an adaptive grid-based thresholding method is applied to the restored image $\hat{f}\left(x,y\right)$.

The restored image is first partitioned into local grids $G_{k}$. For each grid, the local mean and standard deviation are calculated, and the corresponding adaptive threshold is defined as

(11) $$T_{k}=\mu_{k}+\alpha \sigma_{k}$$

where $\mu_{k}$ and $\sigma_{k}$ are the mean and standard deviation of the pixel intensities within $G_{k}$, respectively, and α controls the sensitivity of candidate detection. A smaller α retains weaker responses but may increase false detections, whereas a larger value suppresses the background more strongly but may remove weak bubble-plume responses.

Based on a parameter sweep performed using pre-experimental data that were not included in the final evaluation dataset, α was set to 1.5 and then kept unchanged for all reported leakage conditions. This value is an empirical setting for the present instrument and survey environment and is not interpreted as a universally optimal threshold. The grid dimensions were set to $L_{\theta }=2^{\circ }$ in azimuth and $L_{r}=0.5$ m in range, which are comparable to the characteristic spatial scale of the candidate plume responses observed in the pre-trial data.

Pixels whose intensities exceed the corresponding local threshold are marked as candidate pixels:

(12) $$M\left(p\right)=\{\begin{array}{cc} 1, & \hat{f}(p)>T_{k},(x,y)\in G_{k}\\ 0, & \mathrm{otherwise} \end{array}$$

where M(p) is the binary candidate mask.

The binary map may contain isolated high-intensity pixels, fragmented detections, and elongated reverberation structures. Eight-neighbor connected-component analysis is therefore applied to group spatially adjacent candidate pixels. Let $C_{i}$ denote the ith connected component and $N_{i}$ its number of pixels. Each component is evaluated using physical area, local intensity contrast, shape, and range-continuity criteria.

First, the physical area of $C_{i}$ is calculated as:

(13) $$A_{i}=\sum_{p\in C_{i}}\Delta A_{p}$$

where $\Delta A_{p}$ is the physical area represented by pixel p. For a Cartesian image with uniform spatial sampling,

$$A_{i}=N_{i}\Delta x\Delta y,$$

where Δx and Δy are the Cartesian pixel intervals. For a polar range–angle image, the area of a pixel centered at range $r_{p}$ is approximated as

$$\Delta A_{p}\approx r_{p}\Delta r\Delta \theta ,$$

where Δr is the range interval and Δθ is the angular interval expressed in radians. This range-dependent expression is used when the connected-component operation is performed on the native polar grid.

A component is retained only when

$$A_{i}>A_{min}.$$

Reporting the threshold in physical units avoids ambiguity caused by the range-dependent area of polar-grid pixels.

Second, the mean restored intensity of a component is required to exceed its local background by a prescribed ratio:

(14) $$\bar{I_{i}}=\gamma \mu_{bg,i}$$

where

(15) $$\bar{I_{i}}=\frac{1}{N}\sum_{p\in C_{i}}\hat{f}\left(p\right)$$

is the mean intensity of a local neighborhood surrounding $C_{i}$, excluding the component itself, and γ=[XX] is the local-contrast coefficient. This criterion ensures that the retained component has sufficient contrast relative to its immediate background.

Third, elongated reverberation structures are rejected according to the component aspect ratio:

$$\rho_{i}=\frac{a_{i}}{b_{i}}<5,$$

where $a_{i}$ and $b_{i}$ are the major- and minor-axis lengths of the fitted component ellipse, respectively. This criterion reduces extended seabed responses and approximately linear structural clutter. The threshold was selected using the independent pre-trial data and kept fixed during the evaluation experiments.

An additional range-continuity condition is used to remove points that are spatially connected in the binary image but inconsistent with the principal range distribution of the component:

$$\left|r_{j}-\bar{r_{i}}\right|<\eta L_{r}$$

where $r_{j}$ is the range of the candidate point, ${\bar{r}}_{i}$ is the mean range of the corresponding component, $L_{r}$ is the grid extent in the range direction, and η is set to 1.5. Thus, candidate points with large range deviations from the main cluster are discarded. After the above filtering operations, the remaining connected components are regarded as potential bubble-plume regions.

For each detected plume region C, its location relative to the sonar head is estimated using an intensity-weighted centroid. In polar coordinates, the centroid is calculated as

(16) $$r_{c}=\frac{\sum_{(r,\theta )\in C}r\hat{f}\;(r,\theta )}{\sum_{(r,\theta )\in C}\hat{f}(r,\theta )},\theta_{c}=\frac{\sum_{(r,\theta )\in C}\theta \;\hat{f}(r,\theta )}{\sum_{(r,\theta )\in C}\hat{f}(r,\theta )}$$

where $r_{p}$ and $\theta_{p}$ are the range and azimuth of the pixel p. The circular form used for $\theta_{c}$ avoids ambiguity near an angular wrapping boundary. Compared with an unweighted geometric centroid, the intensity-weighted centroid reduces the contribution of weak boundary pixels and provides an estimate of the acoustic center of the detected plume.

The corresponding horizontal coordinates in the sonar-fixed frame are

(17) $$x_{c}=r_{c}\cos\theta_{c},\;\;\;\;\;y_{c}=r_{c}\sin\theta_{c}$$

where $x_{c}$ is the forward distance and $y_{c}$ is the lateral distance relative to the sonar head. Because the forward-looking sonar primarily measures range and horizontal bearing, $\left(x_{c},y_{c}\right)$ represents a two-dimensional sonar-relative estimate rather than a complete three-dimensional plume position.

To transform the sonar-relative estimate into an Earth-referenced navigation frame, the vehicle position, vehicle attitude, sonar mounting orientation, and lever-arm offset must be considered simultaneously. The coordinate transformation is written as:

(18) $$p_{c}^{n}\left(t\right)=p_{v}^{n}\left(t\right)+R_{b}^{n}\left(t\right)\left[l_{s}^{b}+R_{s}^{b}p_{c}^{s}\left(t\right)\right]$$

where

$$p_{c}^{s}=\left[\begin{array}{c} x_{c}^{s}\\ y_{c}^{s}\\ z_{c}^{s} \end{array}\right]$$

is the detected plume position in the sonar coordinate system, $p_{v}^{\;n}$ is the vehicle navigation-reference position in the local navigation frame, $R_{s}^{\;b}$ is the rotation matrix from the sonar frame to the vehicle body frame, $R_{b}^{\;n}$ is the body-to-navigation rotation matrix determined from vehicle roll, pitch, and heading, and $l_{s}^{\;b}$ is the lever-arm vector from the navigation reference point to the sonar head. For two-dimensional forward-looking sonar measurements, $z_{c}^{s}$ is assigned according to the sonar mounting geometry or the adopted horizontal-plane approximation.

The local navigation-frame coordinates can subsequently be converted to latitude and longitude using the survey coordinate reference system. All distances are expressed in meters, attitude angles are converted to radians before the rotation matrices are calculated, and geographic coordinates are reported in degrees only after the coordinate transformation is complete.

Each sonar frame must be associated with the corresponding USBL position and vehicle-attitude record using a common timestamp or an interpolated navigation timestamp. The uncertainty of the Earth-referenced position is affected by at least the following terms:

1. Sonar range resolution and sound-speed uncertainty;
2. Bearing resolution and beamforming error;
3. USBL positioning uncertainty;
4. Vehicle roll, pitch, and heading uncertainty;
5. Sonar mounting-angle calibration;
6. Lever-arm measurement uncertainty; and
7. Synchronization error between the sonar and navigation systems.

Therefore, the transformed position should not be described as “precise geographic localization” without an uncertainty analysis. The localization results (in Section 3) should report either the sonar-relative range and bearing or the Earth-referenced position together with the above uncertainty sources. If synchronized attitude, mounting-offset, and USBL records were not available for a test run, only the sonar-relative position $\left(r_{c}|\theta_{c}\right)$ should be reported.

Finally, geometric and acoustic features are extracted from each retained plume region. The principal features are defined as

(19) $$A_{C}=\sum_{p\in C_{i}}\Delta A_{p}\;I_{max}={max}_{p\in C}\hat{f}\left(p\right)\;\bar{I_{C}}=\frac{1}{N_{C}}\sum_{p\in C}\hat{f}\left(p\right)\;S_{C}=\sum_{p\in C}\hat{f}\left(p\right)\Delta A_{p}$$

where $A_{C}$ is the physical area of the plume component, $I_{max}$ is its peak restored intensity, ${\bar{I}}_{C}$ is its mean restored intensity, $S_{C}$ is the area-integrated acoustic intensity, and $N_{C}$ is the number of pixels in the component.

If the image is represented on a Cartesian grid with uniform physical pixel area, Equation (19) becomes

(20) $$S_{C}=\Delta x\Delta y\sum_{p\in C}\hat{f}\left(p\right)$$

The unweighted pixel sum can be used only when the physical area represented by every pixel is identical or when a dimensionless image-domain descriptor is explicitly intended.

These features characterize complementary properties of the detected acoustic response: $A_{C}$ describes its spatial extent, $I_{max}$ characterizes its strongest local return, ${\bar{I}}_{C}$ represents its average restored intensity, and $S_{C}$ combines acoustic intensity with physical spatial coverage. Their relationships with leakage aperture and pressure are investigated in Section 3 using the complete experimental dataset and the specified statistical fitting procedure.

Because no synchronized mass-flow or volumetric-flow reference measurements were obtained, these acoustic features are not interpreted as direct estimates of leakage rate. They are used only to characterize and compare the acoustic plume responses under the tested aperture and pressure conditions.

### 2.5. Complete Processing Architecture and Workflow

**Figure 8:**
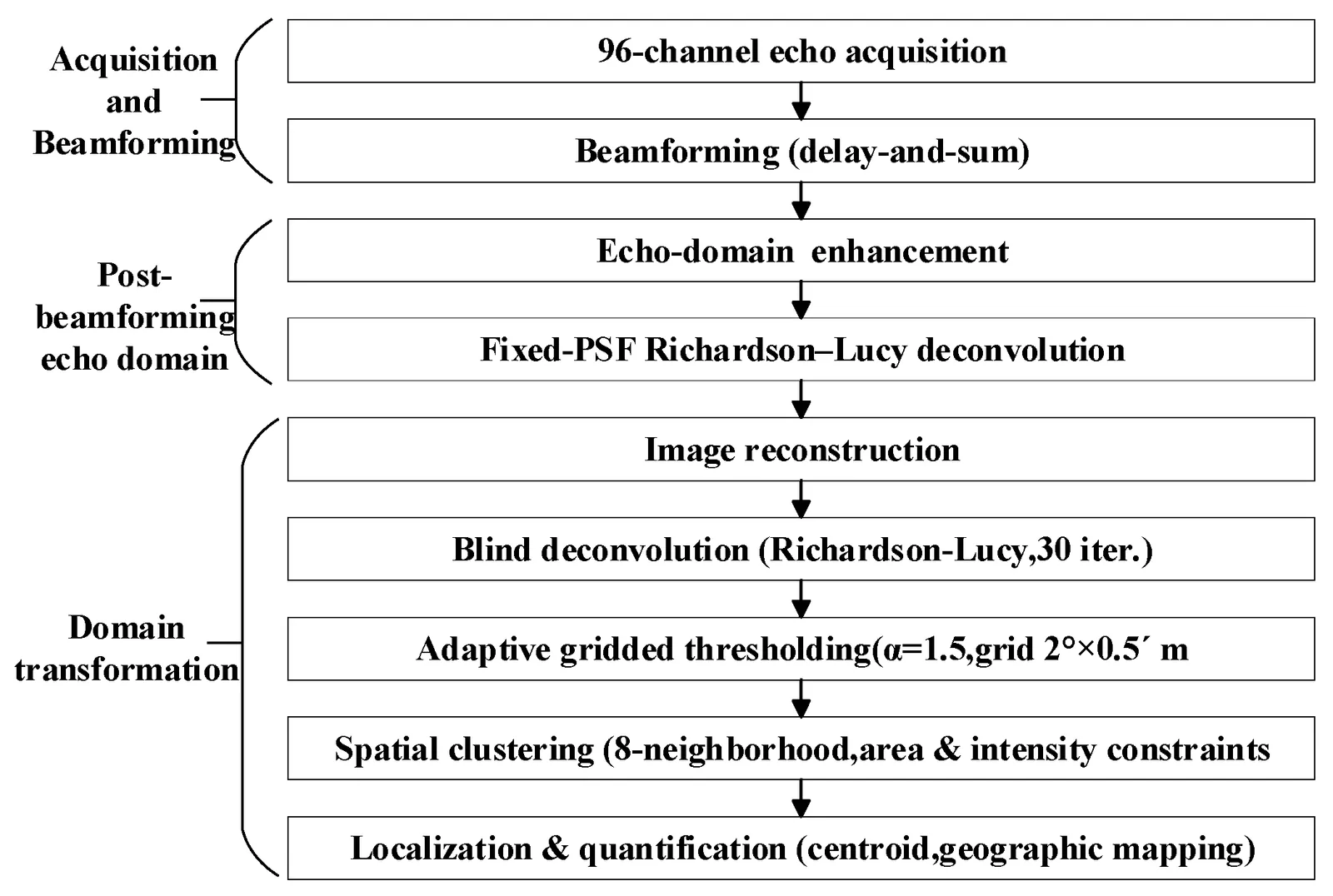
Overall workflow of the proposed echo- and image-domain fusion framework for micro-leak detection, localization, and feature extraction.

The complete workflow of the echo- and image-domain fusion framework is presented in Figure 8. It combines the sonar acquisition system described in Section 2.1 with the processing procedures introduced in Section 2.2, Section 2.3 and Section 2.4. To avoid repeating the detailed mathematical descriptions given above, the workflow is summarized in five stages. The topside processing unit is a standard industrial computer (Intel Core i7, 16 GB RAM), and the software runs on a Windows-based platform.

1. Data acquisition and beamforming: The multichannel array signals are acquired and processed using delay-and-sum beamforming to generate a two-dimensional range–angle acoustic map.
2. Echo-domain enhancement: Local statistical normalization is applied to the beamformed map, rather than directly to the 96 independent raw channels, to reduce spatially varying background responses and retain localized acoustic anomalies.
3. Image-domain restoration: The enhanced map is transformed into an image-domain representation and restored using the blind Richardson–Lucy procedure defined in Equations (7)–(10), in which both the latent image and PSF are iteratively updated.
4. Candidate segmentation and validation: Adaptive grid-based thresholding identifies candidate pixels, and connected-component analysis removes regions that do not satisfy the prescribed physical-area, intensity-contrast, shape, and range-continuity criteria.
5. Localization and feature extraction: The intensity-weighted centroid provides the sonar-relative range and bearing of each retained plume component. Earth-referenced transformation is performed only when synchronized vehicle position, attitude, sonar mounting, and lever-arm information are available. Physical area and acoustic-intensity features are subsequently extracted for quantitative analysis.

As illustrated in Figure 8, the operational workflow operates as a continuous processing pipeline on the topside processing unit, enabling near-instantaneous visualization for ROV navigation. The framework uses statistical information in the beamformed acoustic map before image formation and combines it with structural information obtained during image restoration and segmentation. In this sense, the method differs from an image-only pipeline that operates exclusively on the final displayed sonar image. The effect of each processing stage, however, must be established through the baseline and ablation experiments described in the next Section.

## 3. Experimental Results and Analysis

### 3.1. Sea-Trial Setup and Pre-Deployment

The offshore experiments were conducted in Bohai Bay at a site with a water depth of approximately 14 m and an underwater visibility of approximately 0.5 m. A controlled gas-release device was deployed on the seabed to generate bubble plumes through interchangeable circular apertures. The aperture diameters ranged from 0.5 to 3.0 mm, and the nominal gas-supply pressures ranged from 0.5 to 3.0 MPa at intervals of 0.5 MPa.

The pressure reported in this study was the nominal operating pressure recorded by the field personnel during each release. The pressure immediately upstream of the seabed aperture was not measured independently. In addition, no synchronized mass-flow or volumetric-flow measurements were obtained. Therefore, the aperture diameter and nominal supply pressure are treated as controlled experimental conditions rather than as direct measurements of the gas leakage rate.

Before deployment, the ROV (Shenzhen Dechuang Underwater Intelligent Equipment Co., Ltd, Shenzhen, China) launch-and-recovery system, thrusters, underwater cameras, lighting system, manipulator, communication link, navigation system, multibeam forward-looking sonar, and gas-release device were inspected. After the vessel arrived at the experimental site, the USBL support frame was deployed vertically. The positioning information was monitored from both the ROV control room and the vessel bridge. The multibeam forward-looking sonar was mounted on the ROV, and its operating parameters were configured before data acquisition.

The sonar was operated at a transmission frequency of 700 kHz, with a pulse duration of 0.1 ms and a nominal acquisition-range setting of 200 m. The 200 m value represents the configured acoustic acquisition window and should not be interpreted as the experimentally verified detection range of the bubble plume.

**Figure 9:**
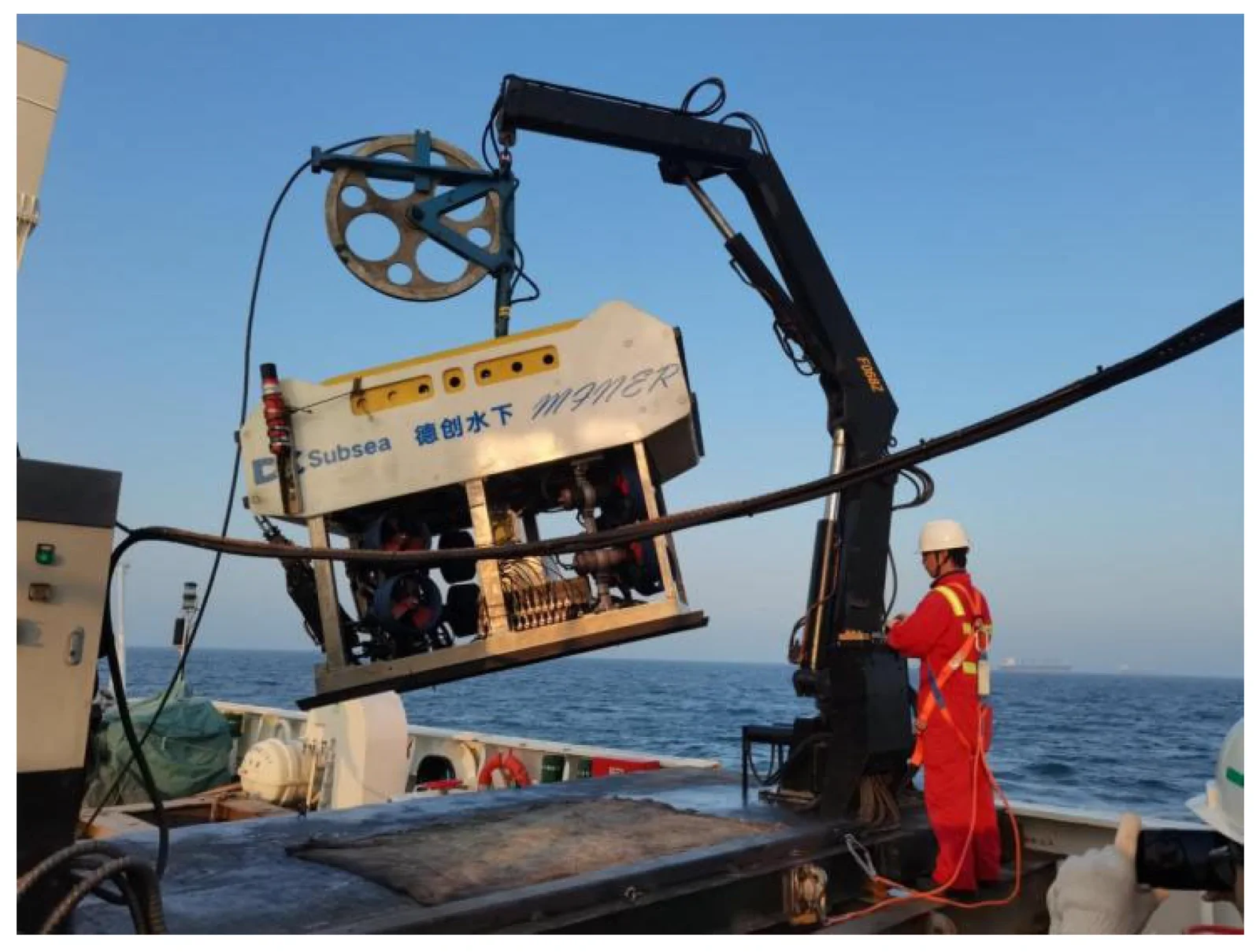
Photograph of the field operation (ROV deployment).

Figure 9 shows the offshore deployment configuration of the ROV-mounted sonar and the controlled seabed gas-release device.

For each experiment, the gas-release device was placed on the seabed, and its position was recorded. The ROV was initially positioned more than 500 m from the release device and then approached the target area at a speed of approximately 0.5 knots while continuously acquiring sonar data. When the operator first confirmed a plume-related response in the sonar image, the ROV position and the corresponding sonar-to-release-device distance were recorded. The ROV continued approaching until the released bubbles could be visually confirmed using the onboard camera.

The distance reported in this study is therefore defined as the field-recorded plume-observation distance during the ROV approach. It is not a probability-of-detection threshold obtained from repeated measurements at systematically controlled stand-off distances. Similarly, the ROV patrol distances exceeding 500 m are cumulative vehicle travel distances and should not be interpreted as acoustic plume-observation distances.

A total of 46 independent controlled gas releases were conducted between 8 and 11 July 2025. Each release and its corresponding ROV approach process were treated as one independent experimental unit. The aperture and nominal-pressure conditions were reset for each release. Among the 46 experiments, 30 contained complete acoustic images suitable for detailed image analysis. These 30 image datasets comprised experiments across six aperture sizes, with the number of tests per aperture ranging from 3 to 11 (see Table 2 for the detailed distribution).

Table 3 presents representative ROV patrol trajectories. The distances listed in Table 2 represent cumulative ROV trajectory lengths calculated from the navigation records rather than instantaneous plume-observation distances.

Table 2 presents the complete field records for the 46 independent gas-release and ROV-approach tests. The table includes aperture diameter, nominal pressure, recorded plume-observation distance, and experimental date.

The column previously described as the “sonar-detected leakage-image coordinate” represents the position of the sonar platform when the plume was first confirmed. It is not an independently estimated position of the leakage source. It is therefore reported as the sonar position at the first confirmed plume observation.

All coordinates are expressed in meters using the same projected coordinate system. The horizontal coordinates were based on the China Geodetic Coordinate System 2000 and a Universal Transverse Mercator projection. In the field navigation records, the first coordinate component denotes northing and the second denotes easting.

The navigation system and sonar acquisition system were synchronized using the common timing signal transmitted by the vessel-mounted GPS-RTK system. This common clock provided the time reference used to associate sonar observations with the corresponding ROV navigation records. Although this arrangement reduced clock inconsistency between the systems, the residual synchronization uncertainty was not independently measured during the experiments.

The numbers of transmitted pings and acquired frames were not recorded separately during the sea trial and cannot be recovered from the available field records. Accordingly, temporally adjacent sonar frames obtained during the same release and ROV approach were regarded as repeated observations within the same experimental unit rather than statistically independent samples. The statistical unit used in this study is therefore the independent release–ROV approach process, and no frame-level statistical significance is claimed.

All offshore experiments were conducted in compliance with Chinese maritime regulations under a formal operation permit, in collaboration with a major offshore oil company. The released gas was compressed air (no hydrocarbons or toxic substances), supplied continuously by an onboard compressor; the total release volume remained within permit limits. On-site safety measures included a designated safety officer, communication with the partner company’s safety team, personal protective equipment, and pre-dive subsea connection inspections. Emergency procedures covered compressor shutdown, ROV recovery, and reporting channels to the vessel master and emergency coordinator. No incidents occurred during the trial.

### 3.2. Detection Results and Acoustic-Feature Analysis

**Figure 10:**
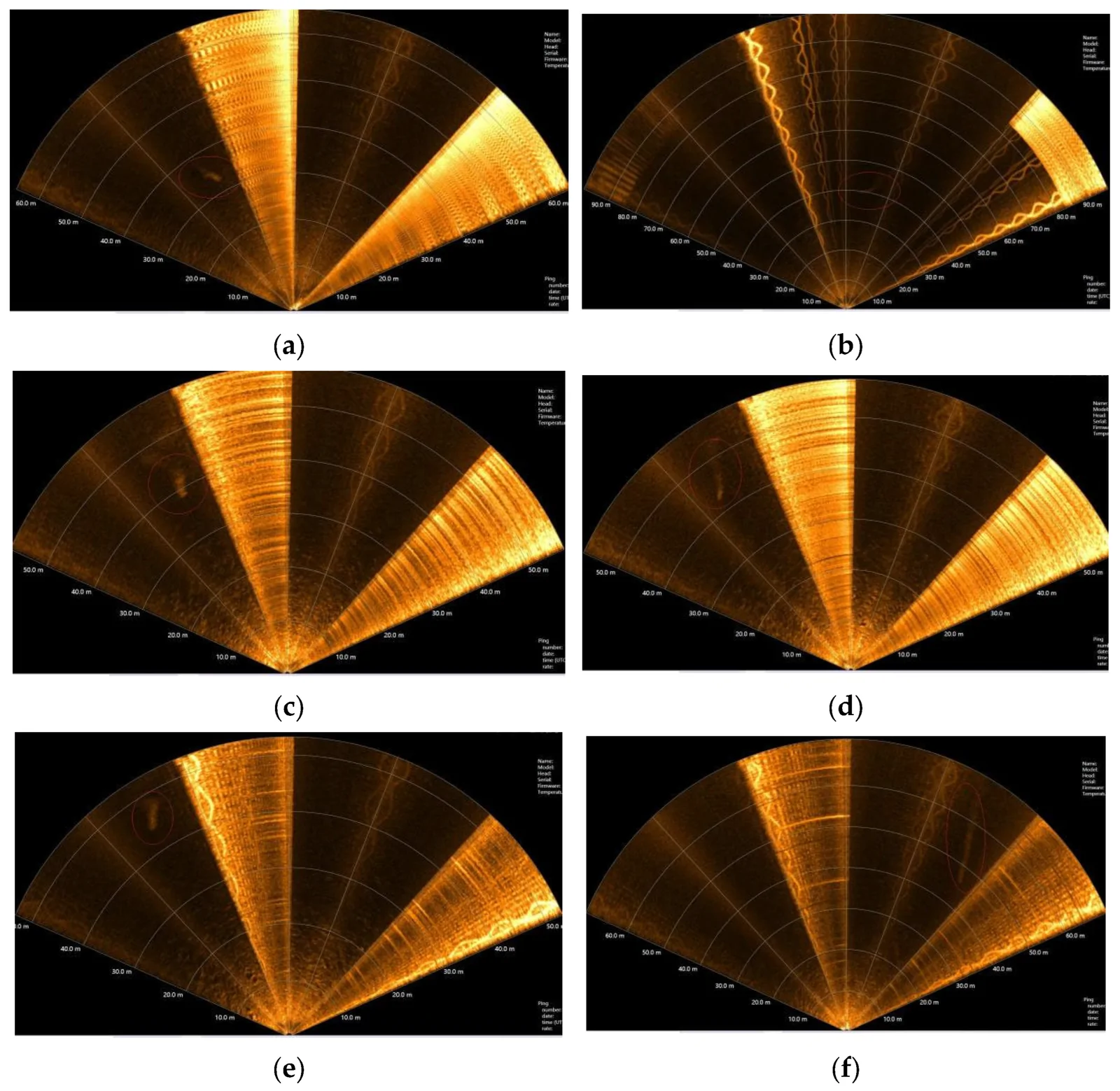
Test results for 0.5 mm aperture: (**a**) 0.5 MPa; (**b**) 1 MPa; (**c**) 1.5 MPa; (**d**) 2 MPa; (**e**) 2.5 MPa; (**f**) 3 MPa.

The 96-channel element-level signals were first beamformed to generate two-dimensional range–angle maps. The temporal-statistical enhancement described in Section 2 was then applied to the post-beamforming range–angle data rather than directly to the raw 96-channel element signals. The enhanced range–angle maps were transformed into Cartesian images and subsequently processed using the blind Richardson–Lucy restoration described in Section 2.3 (in which both the latent image and the point spread function are updated iteratively), followed by adaptive segmentation, connected-region grouping, and candidate filtering. This formulation is therefore consistent with the blind deconvolution procedure presented in Section 2.3, rather than a fixed-PSF deconvolution. Unless otherwise stated, the reported images were processed using 30 Richardson–Lucy iterations, an adaptive-threshold coefficient of α=1.5, and a minimum connected area of five grid cells. These parameters were applied consistently to the analyzed release and background data and were not adjusted separately for individual frames. Test results for 0.5 mm aperture at different pressure are shown in Figure 10.

At the minimum tested condition of a 0.5 mm aperture and a nominal pressure of 0.5 MPa, the plume response was weak and spatially limited but remained distinguishable from the adjacent acoustic background in the selected observation. The corresponding projected plume area was approximately $0.08\;m^{2}$, the relative mean image intensity was approximately 8.2 dB above the local image background, and the recorded plume-observation distance was approximately 32 m.

**Figure 11:**
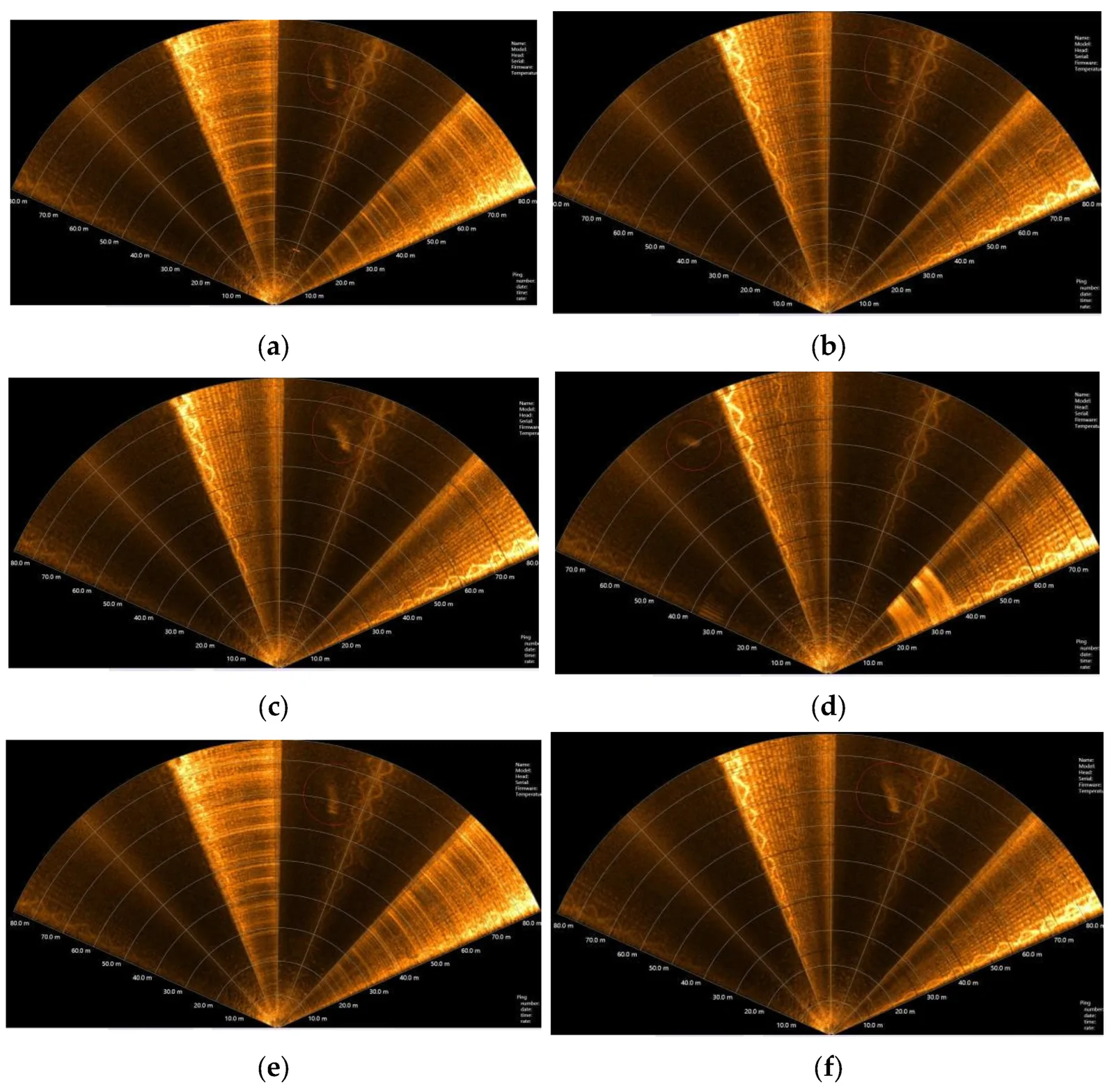
Test results for 2.5 mm aperture: (**a**) 0.5 MPa; (**b**) 1 MPa; (**c**) 1.5 MPa; (**d**) 2 MPa; (**e**) 2.5 MPa; (**f**) 3 MPa.

For the selected 0.5 mm result at a nominal pressure of 3.0 MPa, the projected plume area was approximately $0.35\;m^{2}$; the relative mean image intensity was approximately 16.7 dB, and the recorded plume-observation distance was approximately 47 m. These representative observations show a more spatially continuous and prominent plume response at the higher nominal pressure. However, they do not by themselves establish a universal pressure–response relationship. Test results for 2.5 mm aperture at different pressure are shown in Figure 11.

At a nominal pressure of 0.5 MPa, the selected plume result had a projected area of approximately $0.25\;m^{2}$, a relative mean image intensity of approximately 14.5 dB, and an observation distance of approximately 66 m. At 3.0 MPa, the selected result had a projected area of approximately $0.85\;m^{2}$, a relative mean image intensity of approximately 23.1 dB, and an observation distance of approximately 66 m.

The reported relative intensity was calculated as:

(21) $$I_{rel}=10\log_{10}\left(\frac{{\bar{I}}_{plume}+\varepsilon }{{\bar{I}}_{bg}+\varepsilon }\right)$$

where ${\bar{I}}_{plume}$ is the mean image intensity within the identified plume region, ${\bar{I}}_{bg}$ is the mean intensity within an adjacent local background region, and ε is a small positive constant. These values describe relative plume-to-background image contrast and should not be interpreted as calibrated sound-pressure levels, target strengths, volume-backscattering strengths, or gas-flow measurements.

The complete field records show that the recorded observation distance did not increase monotonically with either aperture diameter or nominal pressure. The descriptive ranges and means for the six aperture groups are presented in Table 4.

These values are descriptive summaries of the field records and are not estimates of aperture-specific maximum detection ranges. The numbers of independent releases differed among the aperture groups, while the ROV trajectory, vehicle attitude, target aspect, plume orientation, water current, and bubble distribution were not controlled independently.

Repeated releases under the same aperture and nominal-pressure conditions also produced different plume-observation distances. For example, the two releases conducted using a 0.5 mm aperture at 0.5 MPa produced recorded distances of 32 and 47 m. This variation indicates that aperture diameter and nominal pressure alone are insufficient to determine the field-observation distance.

Consequently, the available observations are not used to establish a general power-law relationship among aperture diameter, nominal pressure, plume area, image intensity, and observation distance. No synchronized mass-flow, volumetric-flow, near-aperture pressure, or bubble-size measurements were obtained. The extracted image features therefore characterize acoustic plume responses under the tested conditions but cannot be converted into quantitative gas leakage rates.

The 0.5 mm aperture at a nominal pressure of 0.5 MPa represents the minimum condition examined and identified in the present experiments. It should not be interpreted as the absolute sensitivity threshold of the system.

### 3.3. Qualitative Ablation Comparison

The proposed framework combines temporal-statistical enhancement of the post-beamforming range–angle data with subsequent image-domain restoration, segmentation, and candidate identification. To illustrate the contribution of the temporal-statistical processing stage, three images generated from the same representative plume observation were compared using: (1) the original beamformed image; (2) image-domain processing alone (including the blind Richardson–Lucy restoration, segmentation, and clustering); and (3) the complete framework.

In the image-only result (b), the plume response remains identifiable but appears fragmented, with residual background fluctuations of comparable intensity that reduce the plume-to-background contrast. In the combined result (c), the same plume region exhibits visibly higher spatial continuity and a more uniform interior response, while the surrounding background is more effectively suppressed. Based on the image pixel intensities displayed in the common color scale, the peak contrast between the plume region and the adjacent background is noticeably higher in (c) than in (b). In addition, the retained connected region in (c) contains approximately twice the number of pixels within the same plume boundary, indicating improved spatial coherence of the detection.

In the image-only result, the plume-related response remained identifiable, but residual background fluctuations reduced its contrast and spatial continuity. In the combined result, the plume response was more clearly separated from the surrounding background, and the retained region appeared more spatially continuous. The comparison therefore illustrates the contribution of temporal-statistical enhancement to background suppression and weak-plume visualization for this representative observation.

**Figure 12:**
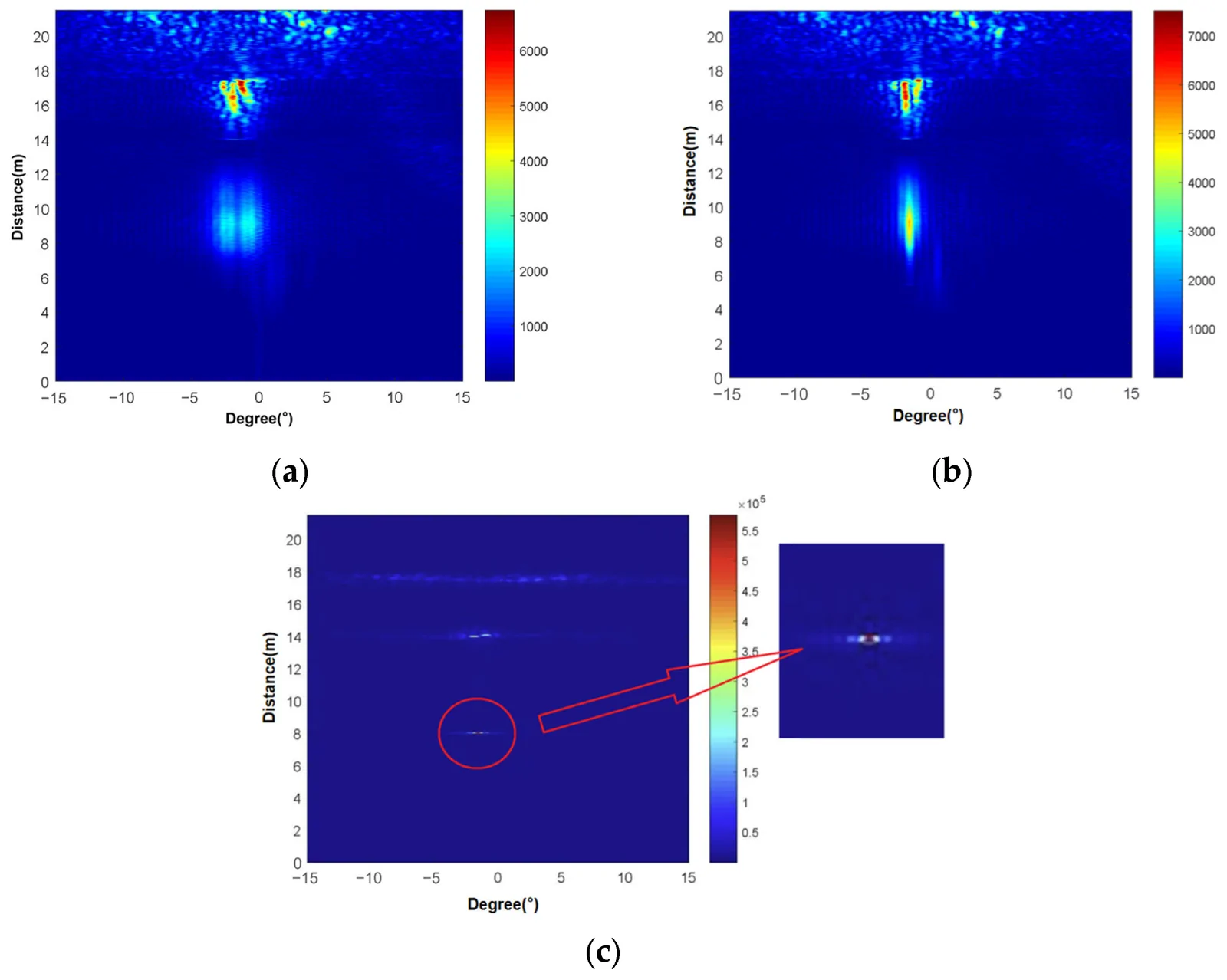
Qualitative ablation comparison using the same representative plume observation: (**a**) original beamformed acoustic image; (**b**) plume-identification result obtained using image-domain processing alone; and (**c**) result obtained using the combined temporal-statistical and image-domain processing framework. Identical spatial coordinates, color maps, color-scale limits, and image-domain processing parameters are used in all three panels.

However, Figure 12 is a qualitative ablation comparison. Complete frame-level annotations and quantitative baseline metrics, such as precision, recall, intersection over union, plume-to-background contrast statistics, and false-positive area, were not available for the full dataset. The figure therefore does not demonstrate statistically significant or universal superiority of the combined method over image-domain processing alone.

The same limitation applies to the processing parameters. The selected values of 30 Richardson–Lucy iterations, α=1.5, and a minimum connected area of five cells produced stable results for the reported experimental data, but a complete parameter-sensitivity experiment was not performed. Environments with different seabed reverberation, biological scattering, suspended particles, or sonar configurations may require parameter verification or retuning. In particular, the absence of false-positive events in the available background sequence should not be used to claim that α=1.5 will produce the same result at another site.

### 3.4. Position Association, Background Evaluation, and Limitations

The ROV navigation records, release-device coordinates, and sonar observations were associated using timestamps referenced to the common timing signal transmitted by the vessel-mounted GPS-RTK system. The sonar position recorded at the first confirmed plume observation was then compared with the known release-device position to calculate the corresponding field-observation distance.

The coordinate pair recorded at the first confirmed plume observation represents the position of the ROV-mounted sonar, not an algorithmically estimated leakage-source position. Therefore, the separation between this coordinate and the release-device coordinate is reported as the sonar-to-release-device observation distance. It is not treated as a localization error.

Although the common GPS-RTK clock provided a consistent time reference, a complete localization uncertainty analysis would additionally require several components that were not available. These include the time-varying ROV heading, roll, and pitch; the sonar mounting orientation; the lever-arm offset between the sonar and the navigation reference point; USBL uncertainty; sound-speed uncertainty; and the residual timing error. These components were not recorded with sufficient completeness for retrospective uncertainty decomposition.

Accordingly, the present study does not report a numerical localization-accuracy value. The coordinate records demonstrate that the sonar observations could be associated with the corresponding ROV and release-device positions during field operation, but they are not used to claim validated source-localization accuracy.

Background performance was evaluated using a continuous 30 min sonar sequence acquired without intentional gas release. The same fixed processing settings used for the release experiments were applied to the background sequence. A false-positive plume event was defined as a background response that passed the complete temporal enhancement, restoration, segmentation, connected-region grouping, spatial filtering, and candidate-selection procedure and was retained as a plume candidate.

No false-positive plume event was observed during the analyzed 30 min non-release sequence. Consecutive frames in this sequence were temporally correlated, and the numbers of pings and frames were not recorded. Therefore, the result is reported only as:

No false-positive plume event was observed during the analyzed 30 min non-release background sequence.

This observation does not establish a zero false-alarm probability. The available sequence represents one site, one limited observation period, and a restricted range of environmental conditions. A statistically supported false-alarm rate would require longer background observations from multiple locations and environmental conditions, with the numbers of pings, frames, and independent background intervals recorded explicitly.

The principal limitations of the offshore evaluation are summarized as follows:

1. The numbers of transmitted pings and acquired frames were not recorded and cannot be recovered from the available field records. Each independent release and corresponding ROV approach was therefore treated as one experimental unit, and no frame-level statistical significance is claimed.
2. The three-panel comparison using the same representative observation showed that the combined temporal-statistical and image-domain processing method produced a clearer and more spatially continuous plume response than image-domain processing alone.
3. The recorded plume-observation distances were obtained during ROV approach experiments rather than controlled probability-of-detection experiments at predetermined stand-off distances.
4. The coordinate at the first confirmed plume observation represents the sonar-platform position rather than an algorithmically estimated leakage-source position. No numerical localization-accuracy claim is made.
5. Although sonar and navigation timestamps used the common vessel GPS-RTK clock, the complete vehicle-attitude, mounting-offset, USBL-uncertainty, and residual synchronization information required for a localization uncertainty budget was unavailable.
6. No synchronized mass-flow, volumetric-flow, near-aperture pressure, or bubble-size measurements were obtained. Quantitative leakage-rate estimation is therefore outside the scope of the present experiments.
7. The 30 min background sequence is insufficient to establish a universal false-alarm probability.
8. A complete parameter-sensitivity analysis and computational benchmark on specified CPU/GPU hardware were not available. Consequently, universal parameter robustness and quantitatively verified performance are not claimed.

To illustrate the complete localization chain described in Section 2.4, we take one representative observation (Test No. 6: 0.5 mm aperture, 0.5 MPa, 32 m) as an example. From the processed sonar frame, the intensity-weighted centroid was estimated as (r_c = 32.0 m, θ_c = −3.5°) using Equation (16), yielding sonar-fixed Cartesian coordinates (x_c, y_c) = (31.94, −1.95) m via Equation (17). This sonar-relative position was then transformed into the navigation frame following the rotation sequence in Equation (18). The transformation used the synchronized vehicle position (118.123456° E, 38.654321° N), vehicle attitude (heading 45°, pitch 2°, roll −1°), sonar mounting offsets (yaw 0°, pitch −3°), and lever-arm compensation (0.5, 0.2, −0.3) m. The estimated target position deviated from the surveyed release-device position by approximately 4.2 m in this single instance. This single-sample deviation is presented solely to demonstrate the complete computational workflow. It does not represent a validated localization accuracy. The full uncertainty budget—including time-varying attitude, mounting calibration residuals, USBL measurement noise, sound-speed variability, and synchronization errors—could not be reconstructed from the available field records. A theoretical decomposition of these uncertainty sources—including sonar range/bearing resolution, USBL positioning, attitude errors, mounting calibration, and lever-arm errors—indicates that the overall horizontal uncertainty is dominated by the USBL contribution (estimated 2–5 m in shallow water). This substantially exceeds the sonar’s intrinsic precision (<0.5 m at 32 m). This explains why the field-observation distances reported in Table 2 are presented as range metrics rather than validated localization errors.

Overall, the offshore experiments demonstrate the initial feasibility of using an ROV-mounted multibeam forward-looking sonar and combined temporal-statistical and image-domain processing to detect controlled underwater bubble plumes under the reported Bohai Bay conditions. The results support field detection and qualitative plume characterization, but they do not establish an absolute detection threshold, a verified maximum detection range, calibrated gas leakage-rate estimation, universal algorithmic superiority, validated localization accuracy, a universal false-alarm probability, or full operational deployment readiness.

## 4. Conclusions

This study developed and evaluated an ROV-mounted multibeam forward-looking sonar system for detecting underwater gas plumes under offshore conditions. The processing framework applies temporal-statistical enhancement to the beamformed range–angle data. This is followed by blind Richardson–Lucy restoration (with both the image and the PSF updated iteratively, as detailed in Section 2.3), adaptive segmentation, connected-region analysis, and candidate filtering in the image domain. This framework was designed to improve the visibility and spatial continuity of weak plume responses in the presence of seabed reverberation and background interference.

Offshore experiments were conducted in Bohai Bay at a water depth of approximately 14 m and an underwater visibility of approximately 0.5 m. A total of 46 controlled gas-release and corresponding ROV-approach experiments were performed using aperture diameters of 0.5–3.0 mm and nominal supply pressures of 0.5–3.0 MPa. Among these experiments, 30 groups contained complete acoustic images suitable for detailed analysis. Bubble plumes were identified under the tested experimental conditions, including the minimum examined condition of a 0.5 mm aperture at a nominal pressure of 0.5 MPa. This result represents the lowest condition evaluated in the present experiments and does not establish the absolute sensitivity threshold of the system.

The recorded plume-observation distances ranged from 32 to 66 m. The longest recorded observation of 78 m should not be interpreted as a verified maximum detection range because the experiments were conducted during continuous ROV approaches rather than at systematically controlled stand-off distances. Likewise, the ROV patrol distances of more than 500 m represent cumulative vehicle travel distances rather than acoustic detection ranges. Although representative results indicated that the projected plume area and relative plume-to-background image intensity generally became more prominent under some larger-aperture or higher-pressure conditions, the observation distance did not vary monotonically with aperture diameter or nominal pressure. The results therefore provide descriptive evidence of plume responses under the tested conditions but do not support a general power-law relationship or quantitative leakage-rate estimation. Such estimation would require synchronized mass-flow or volumetric-flow measurements, near-aperture pressure measurements, and additional bubble-characterization data.

The three-panel comparison using the same representative observation showed that the combined temporal-statistical and image-domain processing method produced a clearer and more spatially continuous plume response than image-domain processing alone. Because complete frame-level annotations and quantitative baseline metrics were unavailable, this result is presented as a qualitative ablation comparison rather than proof of statistically significant or universal algorithmic superiority. In addition, the numbers of transmitted pings and acquired frames were not retained in the available field records. Each controlled release and its corresponding ROV approach were therefore treated as one experimental unit; temporally adjacent frames were not considered statistically independent samples, and no frame-level statistical significance is claimed.

The recorded coordinates at the first confirmed plume observations represent the positions of the ROV-mounted sonar rather than independently estimated leakage-source positions. Consequently, these coordinates were used to determine the field-observation distances but not to calculate localization errors. A numerical localization-accuracy claim is not made because the sample-level estimated source coordinates and the complete information required for an uncertainty analysis, including vehicle attitude, sonar mounting offsets, USBL uncertainty, sound-speed uncertainty, and residual synchronization error, were unavailable. The previously reported value of 8.7 m has therefore been removed from the quantitative conclusions.

No false-positive plume event was observed during the analyzed 30 min non-release background sequence when the same processing parameters were applied. This observation is limited to the available sequence and the environmental conditions encountered during the experiment; it does not establish a zero or universally applicable false-alarm probability. Longer background observations from multiple sites and operating conditions are required for statistically supported false-alarm evaluation.

Overall, the offshore results demonstrate the initial feasibility of the ROV-mounted sonar system and the combined temporal-statistical and image-domain processing framework for detecting and qualitatively characterizing controlled underwater bubble plumes under the reported Bohai Bay conditions. Further work should include controlled range-dependent detection experiments, complete ping and frame records, and quantitative baseline and ablation evaluations. In addition, synchronized flow measurements, sample-level source-localization analysis, longer multi-site background datasets, parameter-sensitivity analysis, and computational benchmarking on specified hardware are also needed. These investigations are necessary before making broader claims regarding maximum detection range, quantitative leakage-rate estimation, validated localization accuracy, universal algorithmic superiority, performance, or operational deployment readiness.

## Figures and Tables

**Table 1:** Comparison of representative methods for subsea gas leak detection and related acoustic processing.

Study	Method	Processing Domain	Target Type	Key Limitation Gap
Leighton [1]	Acoustic bubble theory	Theoretical	Bubble dynamics	Not applied to leak detection
Yang et al. [4]	Passive acoustic bearing estimation	Signal (passive)	Gas leaks	Passive only; no imaging or localization
Gu et al. [6]	Automatic object detection	Image domain	Underwater objects	Not specific to gas leaks
Ge et al. [9]	Deep learning (CNN)	Image domain	Sonar targets	Requires labeled training data
Liu et al. [10]	CNN + Transformer + HOG fusion	Image domain	Sonar image segmentation	Supervised; requires training
Hong [11]	Robust detection method	Image domain	Sonar targets	Image-domain only
Cao et al. [12]	MBES image classification	Image domain	Gas leakage flow	Image-domain only; no echo-domain processing
Zhang et al. [14]	AUV-based pipeline tracking	Image domain	Pipeline tracking	Focused on tracking, not leak detection
Rao et al. [15]	Blind sonar image super-resolution	Image domain	Sonar image quality	Image restoration only
Urban et al. [18]	Echo grid integration	Echo domain (pre-beamforming)	Bubble quantification	Statistical processing only; no image-domain structural analysis
This work	Echo-image dual-domain fusion	Echo + Image domains	Gas micro-leaks	Unsupervised; no training data; 32–66 m observation

**Table 2:** Complete field records of the 46 controlled gas-release experiments.

No.	Aperture Diameter (mm)	Nominal Pressure (MPa)	Observation Distance (m)	No.	Aperture Diameter (mm)	Nominal Pressure (MPa)	Observation Distance (m)
1	1	1.5	62	24	3.0	1.0	62
2	1	1.5	78	25	3.0	0.5	62
3	1.5	1	75	26	3.0	1.5	62
4	1.5	1.5	75	27	3.0	2.0	66
5	1.5	0.5	65	28	3.0	2.5	70
6	0.5	0.5	32	29	3.0	3.0	70
7	0.5	1.0	40	30	2.5	2.0	65
8	0.5	1.5	43	31	2.5	2.5	66
9	0.5	2.0	45	32	2.5	3.0	66
10	0.5	2.5	46	33	2.5	1.5	66
11	1	0.5	67	34	2.5	1.0	70
12	1	1.0	67	35	2.5	0.5	66
13	1	1.5	75	36	2.0	0.5	60
14	1	2.0	75	37	2.0	2.5	60
15	2.0	0.5	45	38	2.0	2.0	62
16	2.0	1.0	58	39	2.0	1.5	62
17	2.0	1.5	66	40	2.0	1.0	62
18	2.0	2.0	45	41	0.5	1.5	50
19	2.0	2.5	45	42	0.5	2.5	49
20	2.5	0.5	55	43	0.5	3.0	47
21	2.5	1.0	54	44	0.5	2.0	47
22	2.5	1.5	55	45	0.5	1.0	47
23	3.0	1.0	78	46	0.5	0.5	47

**Table 3:** The results for three representative test runs.

Test	Starting Point Coordinates (X, Y)	Leak Point Coordinates (X, Y)	Patrol Distance (m)
1	(4,317,476.29, 336,002.28)	(4,317,830.09, 336,443.38)	565
2	(4,318,226.87, 336,483.09)	(4,317,715.44, 336,391.48)	520
3	(4,318,014.05, 336,827.20)	(4,317,690.27, 336,373.91)	557

**Table 4:** The descriptive ranges and means for the six aperture groups.

Aerture (mm)	Independent Tests	Recorded Distance Range (m)	Mean Distance (m)
0.5	11	32–50	44.8
1.0	6	62–78	70.7
1.5	3	65–75	71.7
2.0	10	45–66	56.5
2.5	9	54–70	62.6
3.0	7	62–78	67.1

## Data Availability

Data are contained within the article.

## Abbreviations

The following abbreviations are used in this manuscript:

ROVs	Remotely Operated Vehicles
AUVs	Autonomous Underwater Vehicles
